# Deploying Spatial Data for Coastal Community Resilience: A Review from the Managerial Perspective

**DOI:** 10.3390/ijerph18020830

**Published:** 2021-01-19

**Authors:** Xiameng Huang, Yanqing Song, Xuan Hu

**Affiliations:** 1 School of Navigation Engineering, Guangzhou Maritime University, Guangzhou 510725, China; xiamengh123@163.com; 2 School of Public Affairs, Chongqing University, Chongqing 400044, China; mango08@cqu.edu.cn

**Keywords:** keyword coastal community, resilience, spatial data, decision-making, sustainability

## Abstract

The use of spatial data for coastal community resilience applications has diversified as a consequence of the increasing availability of data, and extensive development in data processing. However, the true value of spatial data is not fully exploited as a result of lacking scientific managerial models that incorporate spatial data into decision-making. This article synthesizes the cross-disciplinary literature review on deploying spatial data for coastal community resilience from the managerial perspective. It systematically reviews research addressing the topic of deploying spatial data for coastal resilience operations from the earliest available to 1999. The review uses 142 studies to address three research questions: (1) What kind of data can be obtained for coastal resilience situational awareness? (2) What outcomes have spatial data attributed to coastal resilience applications? and (3) What are the missing pieces (gaps) in connecting the spatial data with coastal resilience applications? In addressing these research questions, the authors review articles based on three dimensions including the availability of spatial data, the availability of applications, and limitations. Based on the findings of the analysis, the authors conclude that the managerial perspective of deploying spatial data in coastal hazards are understudies, and outline problem formulation, mission prioritization, and information salience as an agenda for future research.

## 1. Introduction

In the past decades, the coastal communities in the United States have sustained tremendous damages as a result of several severe coastal storm events such as Hurricane Katrina (2005), Hurricane Sandy (2012) and Hurricane Harvey (2017). These extreme events have led to substantial loss of livelihoods, costing billions of dollars in the form of destroyed private and public property and mobilization of emergency response personnel and resources. Volumes of scientific evidence and data suggest extreme weather events will continue to multiply and intensify. Nevertheless, it seems that the resilience of natural systems in coastal communities are prone to worsen in facing threats from climate change and sea-level rise and evolving societal pressures from the growing coastal population and needs for construction development [1]. Without actions and investment to strengthen the resilience of communities, these extreme events will continue to drain federal, state, and local budgets, hurt businesses’ bottom lines and threaten the prosperity of future generations. To this end, improving community resilience is demanding.

To improve community resilience, situational awareness information is a necessity. This is because extreme coastal events are more unique than history is averaging. Take Hurricane Sandy as an example—the observed surge is estimated to occur every 400–800 years [2] or even over 1000 years [3]. Such low probability events are difficult to predict based on historical data. In light of this, analysis of spatial data sets offers tremendous opportunities in improving community resilience by better and more timely situational awareness.

Despite such opportunities, scholars have recently questioned the extent to which geospatial data is capable of fulfilling the need for disaster management. Put differently, the importance of incorporating spatial data in disaster response has been repeatedly emphasized in literature [4,5,6,7]; operational-wise, the implementation is sparse during the disaster management in practice [8]. This is because, on the one hand, spatial data is large in volume, varied in format, and has high velocity in data streams, adding to the difficulties in collecting, storing, and processing these spatial data. On the other hand, hazard response is time sensitive which in turn requires timely information. The two conflicts, heavy in computation and emergent in information demand, combine to have limited the usage of spatial data in practice. While many works address the research topic of deploying spatial data for functions such as situational awareness, vulnerability analysis, and sentiment analysis, how to handle the large data sets on time remains challenging. The reality is that while we are capable of deploying a growing number of spatial sensing technologies for data collection during natural disasters, the vast quantities of collected data still have to go through painstakingly manual analyses, and crucial information can often no way be extracted from these data sets in time to support critical decision-making. Therefore, in addition to the data processing algorithms’ development, coordination and integration of spatial data with decision-making processes remains sparse.

In response, this article focuses on investigating the capability of spatial data in community resilience applications from a managerial perspective. This review is not intended to be comprehensive, but it is selective in the sense that it will focus on issues on the coordination and integration of spatial data for coastal resilience decision support. First, we selected coastal resilience as the scope of this review, and hurricane/typhoon is selected as the main hazard. Compared to other hazards such as the earthquake that often lasts less than 60 s, hurricanes form and grow in a much longer period, which squeezes time for spatial data to step in to assist decision-making. Furthermore, this review is performed based on a managerial perspective. Put differently, our main interest is not to investigate the development of spatial data algorithms, but focuses on the managerial perspective of deploying spatial data—how to coordinate and integrate spatial data to support disaster management.

To set an agenda for future research, we present a cross-disciplinary systematic literature review of using spatial data for coastal community resilience decision support. It is deemed that the insights provided by this research help identify where gaps exist in connecting the state of art spatial data to the practical decision-making in coastal resilience applications. The analysis of this review is guided by three research questions

1. What kind of data can be obtained for coastal resilience situational awareness?
2. What outcomes have spatial data attributed to coastal resilience applications?
3. What are the missing pieces (gaps) in connecting the spatial data with coastal resilience applications?

To answer these research questions, we adhere to the Preferred Reporting Items for Systematic Reviews and Meta-Analyses (PRISMA) [9]. The review yielded 142 studies. Based on our analysis, we summarized the capability of different forms of spatial data and how these forms of spatial data fit into the decision-making process in coastal resilience applications. We then highlighted gaps connecting the spatial data with decision-making in coastal resilience. Based on these findings, we concluded by outlining a future research agenda.

## 2. Methodology

In these studies, three strategies were used to identify eligible articles. The first choice made was to include only published journal articles, because journal articles have been through a review process that acts as a screen for quality [10]. Therefore, we used the ISI Web of Science to conduct our literature search. The research period was expanded from the earliest available data up to 30 December 2019. The keywords we used consist of two parts: data type and field type. In the data type, “spatial data”, “LiDAR data” (and other forms such as laser scanning”,” imagery”, “GIS”, “user generated information” are set as the search terms. Regarding the field, we used “hurricane”, “typhoon”, “storm surge”, “coastal hazard” as searching keywords. We applied the AND logic operation between the data type and field type and OR logic operation within each part when filtering the literature. This was done to ensure that a broad spectrum of research on spatial data was included for consideration. This search generated more than 1590 studies; it was last conducted in December 2019.

The second choice made was to identify work related to the decision-making process. Among the 1590 studies, according to the Web of Science, 1566 studies are attributed to science and technology while only 655 articles are related to social science. As explained earlier, the focus of this review is not on a technology perspective, but on a managerial perspective, thus we further eliminated irrelevant articles by a full reading of abstracts.

### 2.1. Eligibility Criteria 

Topic: Abstracts of articles included any of the following terms: “assessment”, “evaluation”, “management”, “analysis”. It is important to note that terms such as “modeling”, “monitoring”, “detection”, “mapping” were not included for analysis because these terms are related to the technology perspective rather than the managerial perspective. Narrowing our search in this way helped to focus on the managerial perspective of using spatial data.

Publication year: Studies that were between the earliest available and 2019 were included.

Language: Only articles written in English were eligible for incorporation.

Publication status: Only international peer-reviewed journal articles from the Web of Science Core Collection were considered eligible. We acknowledge that some books have been highly important in our analysis. However, we would need to exponentially increase our time to review these books whereas the methods are often introduced in journal articles; thus we determined to exclude books in our review analysis.

### 2.2. Review Method

**Figure 1:**
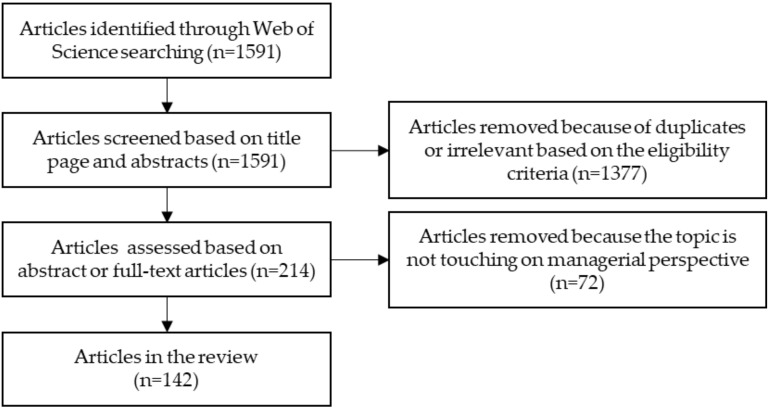
PRISMA Flowchart for database searches and retaining studies.

Following the literature search, a total number of 1591 studies were identified. We then engaged in a selection process following the steps outlined by Liberati, Altman [11] and the flowchart for our review were illustrated in Figure 1.

Following the PRISMA screening process, studies were screened by scanning the article title and abstracts based on the eligibility criteria. Those irrelevant and duplicate articles were removed.

In the second step, we scanned the articles again by reading the full abstract and the full text if necessary. In this step, studies that are not directly touching managerial perspective or spatial data were removed. Following this screening process, 142 studies remained and were included in the systematic review.

For each study in the systematic review list, we developed an information extraction form to summarized articles according to the following criteria: authorship, publication year, title, data source, and data type as outlined in Table 1. 

## 3. Results

A total of 142 articles were included in the systematic review. Among them, 89 articles were based on the GIS platform; the articles related to LiDAR data, remote sensing imagery data, and the user-generated information or crowdsourcing were 27, 17, and 9 respectively. It is worthwhile mentioning that each article only counted once in the type. For instance, in an article that incorporates different spatial data such as LiDAR or imagery into the GIS for analysis, we attributed this article as GIS type and it is not counted in the LiDAR or imagery articles. In all, it is reported that coastal hazards are increasingly observed and monitored using a loosely coupled network of geospatial sensors. Different data acquisition and processing technologies are often deployed to gather information. In the following, the systematic review results will be introduced based on the availability of data, the availability of applications, and the availability of managerial tools.

### 3.1. The Availability of Data

In all, the identification of suitable data is the prerequisite for further incorporating them into the decision-making process [12]. Despite the potential of spatial data, the availability of such datasets remains challenging. A summary of different data is depicted in Table 2.

GIS data are ancillary data previously collected and stored in transactional databases. These data provide essential baseline information including but not limited to demographics, elevation data, land use, municipality boundary, flood zone, and so on. Most GIS data are prepared and summarized by federal agencies such as the U.S. Geological Survey (USGS), Federal Emergency Management Agency (FEMA), and state agencies such as the New Jersey Office of Information Technology (NJGIN), which are publicly available. However, these data are static and carefully prepared for more general purposes: they sacrifice processing efficiency for details, and they do not convey real-time situation information about the impacted areas. Therefore, under normal circumstances, these ancillary spatial data are potent in predictive analysis, but not equally effective in invalidation or interactive analyses.

Citizens in the surrounding communities have the quickest access to the disaster, which has a strong potential capability of sensing time-sensitive disaster information [13], contributing disaster information in the form of user-generated information or crowdsourcing. The development of IoTs and social media have provided new paths for the public to share their “voice”. Average citizens even without domain knowledge are able to record disaster situations [14] through different channels as summarized in Table 2. Micro-blogs (e.g., Twitter, Blogger, Weibo) contain rich-in-detail geo-tagged text messages, which are extensively used for situation description [15,16] and sentiment expression [17,18]. In addition, crowdsourcing imagery data from social media (e.g., Facebook, Weibo, WeChat) are playing an increasingly important role during crisis events [19]. Other platforms such as discussion forums (e.g., Quora, Reddit), social gaming sites (e.g., Gree, Mobage), digital content sharing platforms (e.g., Flickr, Instagram, YouTube) provide additional data that can be further explored for coastal community resilience applications’ purposes.

Moreover, many remote sensing techniques are now capable of facilitating the capturing of real-time spatial data. Imagery is one of the most common types of data collected during natural disasters. Real-time or near real-time observations from satellite, aerial, and ground platforms serve as essential means for imagery data collection for situation awareness. Among them, aerial imagery is the primary source [21] because aircraft or drones are often ready to be deployed to capture either vertical or oblique imagery in a timely manner. Meanwhile, oblique aerial imagery often has a higher resolution than the satellite imagery data, making it more accurate for supporting detail visualization [22]. In addressing Hurricane Sandy, the National Assessment of Coastal Change Hazards (NACCH) conduct surveys to provide robust scientific findings that help to identify coastal community resilience including beach morphology change, dune erosion, and sea-level rise. On the other hand, satellite imagery data is most efficient in terms of capturing the terrain condition of a spacious area [23]. Satellite imagery data can be obtained from NASA Earth Observatory and Google Imagery. Since the repeat interval of most satellites is often daily to monthly [24], it is most suitable for monitoring and modeling the relationship between human activity and long-term environmental or climate impact [23]. 

Light Detection and Ranging (LiDAR) is another emerging technology that facilitates the collection of spatial data in a disaster environment [25]. Depending on the difference of the data acquisition system and platform, LiDAR technologies accommodate the needs of mapping both large-scale objects such as terrain [26] and small-scale objects such as buildings and vegetation [27]. Airborne LiDAR mapping has more or less become the routine survey to track the vulnerability of the coastal communities. For instance, for the New Jersey shoreline alone, at least 10 data sets (2012 USGS EAARL-B Pre-Sandy Lidar, 2012 USACE NCMP Post-Sandy Lidar, 2012 USGS EAARL-B Post-Sandy Lidar, 2013 USACE NCMP Topobathy Lidar, 2013 NOAA NGS Topobathy Lidar, 2014 NOAA OCS Topobathy Lidar, 2014 USGS CMGP Lidar, 2014 NJMC Lidar, 2015 USACE NCMP Topobathy Lidar, 2017 USACE NCMP Topobathy Lidar) are available in NOAA digital coast. Besides, other research agencies also collected valuable mobile (2012 Rutgers Post-Sandy Mobile LiDAR, 2014–2016 Rutgers Ocean County Mobile Lidar; 2017 Rutgers Post-Harvey Mobile Lidar.) [28] and terrestrial LiDAR data [20] to trace the coastal community resilience. LiDAR data have multiple benefits. LiDAR uses active sensing mechanisms, eliminating the need for ambient light to operate. Moreover, compared to imagery approaches, the LiDAR system provides data with better spatial accuracy [29,30]. Last but not least, LiDAR data are rich with in-depth information, which has better potential in providing component-level insights.

### 3.2. The Availability of Applications

Spatial data applications are playing an increasingly important role in disaster management and have proven to offer a variety of opportunities to enhance community resilience [31]. Impacts of disasters are geographically located and have geographic addresses [14]. Compared to attribute information obtained from other sources, spatial data will provide rich information with global-wise perception on how the event happens, and to what extent the event has. Spatial data are reliable tools that have been used in predicting, modeling, simulating, evaluating, assessing, and analyzing the geo-environment catastrophes, which overcomes the bottlenecks caused by the conventional attribute data in reflecting the real-time disaster information cost-effectively and accurately. In this section, we perform a literature survey on how different types of spatial data address the four dimensions (built-up, environment, social, economic) of community resilience as depicted in Table 3. As the social and economic dimensions are difficult to directly extract or quantify from the spatial data, thus, we merge these two dimensions.

GIS data inherently cover all dimensions of community resilience (summarized in Table 2), and as a result, they have been naturally extended to further analysis. The value of ancillary geospatial data in resilience analysis is widely recognized such as in flood inundation modeling [32] and disaster operation support [33]. Nonetheless, restricted by the temporal resolution, GIS data fall short in reflecting real-time disaster situations. This barrier has limited the merit of GIS data in real-time scenario analysis. Instead, since many alternatives lack such integration [34], GIS, as a system, has grown as an important decision support tool [35,36,37], especially when a decision involves integrating data from many different structures and sources [14] as well as consideration of situations over time [38]. For instance, SLOSH [39] and HAZUS [40] are two famous GIS-based models to address coastal community resilience. National Weather Service (NWS) integrated the SLOSH model with GIS to visualize storm surge vulnerability, estimate storm surge heights, and to predict hurricanes [39]. FEMA HAZUS along with GIS enables the capability of economic and social losses’ estimation, which allow officials to evaluate, plan for, and mitigate the effects of hurricane winds and surge [41]. There are numerous studies built upon the SLOSH [2] and HAZUS [41]. In addition, other researchers extended the capability of GIS as a Decision Support System (DSS) by proposing a conceptual framework [33,37,42,43], developing systems [35,42], and spatial analysis tools [43,44].

Crowdsourcing data provides a unique channel for the community-based participation through “citizens as sensors” [45,46]. A systematic literature review on using crowdsourcing data for disaster management can be found in Horita et al. [47]. The increasing development of crowdsourcing data, as well as corresponding data analytics tools, has enabled a new bottom-up channel between citizens and public authorities [23]. “Citizens as sensors” is a novel and vital approach to encourage communities to collect, process, and deliver information. Specifically, in Oxendine, Schnebele [48], the authors illustrated how non-authoritative data can supplement traditional data sources by providing valuable information during emergencies. The most significant contribution of crowdsourcing data is that it fills the gap of other objective data (GIS, remote sensing data) and provides a pathway to discover and analyze the social and economic dimensions of community resilience. It empowers authorities or experts to hear from the “people’s voice” through “crowd voting”, which can be extended human activity pattern analysis [38], sentiment analysis [17,49], and crisis communication [50]. For instance, Sakaki, Okazaki [16] argued that the proposed method of using microblogging for earthquake detection is faster than the rapid broadcast of announcements of the Japan Meteorological Agency (JMA). In addition, crowdsourcing or “people’s voice” offers a new dimension of information—that government and authorities are largely absent—to understand the psychological condition of the victims through sentiment analysis [17,18,49]. Despite this, other studies integrated crowdsourcing data as part of the Spatial Decision Support Systems (SDSS) [15,36,51] and developed new machine learning [15,16,49,51] and statistical analysis [51] tools.

Remote sensing data fits right in the context of community resilience applications since it objectively records the morphology change of the earth’s surface over a large area within a short time interval [52]. Imagery data are the primary source and based map for disasters [21]. Carried by different data acquisition platforms (e.g., satellite, UAV), the image data comes with different resolutions and characteristics. Satellites can cover a large-scale area within a short interval of time. Such rapidness contributes to the prompt capture of the earth’s surface such as sediment plume [53] and land surface [54,55]. A thorough review of the status of satellite remote sensing and image processing techniques for mapping natural hazards can be found in Joyce, Belliss [56]. The authors conducted a comprehensive review of using satellite imagery data for different hazards analysis including coastal flooding. Despite this, the advancement in high-resolution imagery sensors also enables the capability for satellite imagery to capture community-level features. For instance, Sohn and Dowman [57] combined satellite imagery data with LiDAR data to extract building information. Compared to satellite imagery, oblique imagery data collected by helicopters or UAVs have proven potential in community-scale damage assessment [58]. Helicopters and UAVs have been increasingly used to generate real-time or near real-time hazard maps [22,59] including land surface buildings and infrastructure. More recent studies investigated the feasibility of advanced image processing algorithms to extract component-level details. For instance, Zhou, Gong [30] proposed a structure from motion- (SFM) based method to reconstruct 3D disaster scenarios from oblique images. Besides built-up and environment dimensions, a recent study by Jean, Burke [60] also proved potential and pointed to a direction of integrating imagery data for social and economic studies.

Besides, LiDAR data provides additional rich-in-detail layers for community resilience applications. It is extensively extended for coastal hazards’ visualization [27], terrain analysis [68], damage assessment [30,58,72,73], features extracted for modeling, simulation, and knowledge finding [74,75]. LiDAR data can be utilized to not only qualify (what imagery data also does) but more importantly, quantify the earth’s surface features and keep track of the time series morphology changes. Popular artifact built-ups that can be quantitatively measured using LiDAR data include but are not limited to building [57,58,62], transportation infrastructure [27,63], hydraulic infrastructures [20,66], and street furniture [64,65]. Besides, other researchers exploit LiDAR in environment mapping such as land surface [67], forest and trees [68,69] and dune and beach [70]. The importance and capability of LiDAR in disaster response are highlighted in many studies [30,58,72,73], and as a result, LiDAR processing has evolved from basic visualization to more advanced change detection, and more advanced computer vision and machine learning-based algorithms. Besides visualization, one of the most easy-to-operate methods for LiDAR-based analysis is change detection. Multiple temporal resolution data sets are geo-referenced and compared to trace the pre-event and post-event differences [76,77]. Other researchers investigated the feasibility of using computer vision-based algorithms [73] and machine learning-based algorithms to segment and classify LiDAR data for more advanced feature extraction [58,69,72]. Similar to imagery data, LiDAR data is often not directly extended to analyze the social and economic impact. One exception is the work by Lwin and Murayama [71], who deploy LiDAR for human population estimation.

### 3.3. Limitation in the Spatial Data

Yet, spatial data is not perfect, and it should be noted that from the technical perspective, there are problems that remain undressed. Table 4 summarizes techniques, strengths, weaknesses, and gaps in decision support of different spatial data. For any spatial data, quality is always worth special concern [34]. These quality issues of spatial data include error propagation in the GIS system [78], credibility in crowdsourcing data [19], and resolution [57,79]. The second problem is how to address time and resource-constrained disaster environments by improving data processing efficiency. Disasters are often characterized by limited resources and response time. Under such circumstances, it is challenging to process spatial data effectively and efficiently. Especially when advanced data processing algorithms (e.g., computer vision-based, machine learning-based) are adopted, the availability of labeled training datasets [80] and scalability of data processing in cloud or other bin computation infrastructures [81] require further investigating. Last but not least, the dynamic change in the environment of coastal hazards urges for more adaptive algorithms such as service-oriented [82,83] or human interaction [84], which require further implementation.

While the potential of spatial data may not be fully exploited from the technical perspective, managerial tools might be good complementation to step in to leverage spatial data in decision-making in coastal hazards. As indicated in Table 4, all types of spatial data are subjected to managerial gaps. From our review analysis, it is evidenced that GIS is the major platform for collecting, storing, and analyzing spatial data in coastal hazard response, as we reported that 89 out of the 142 articles are GIS-related. Specifically, GIS as a system, has grown as an important decision support tool [35,36,37], integrating data from many different structures and sources [14]. GIS can support the decision-making task by providing a framework to locate all spatial data [89]. However, decision-making in practice is not determined by the potential of spatial data or what GIS can offer, but by the intelligence required from the decision-makers’ side. Put differently, while disaster data and the processing algorithms are more abundant than required, the selection of appropriate datasets, criteria, and scale is a critical part of transferring spatial data into intelligence required by the decision-making process, which is not fully incorporated into GIS or other existing platforms.

For instance, Hoque, Phinn [12] argued that effective risk assessment requires the weighting of spatial data processing criteria in the context of decision-making analysis. They further formulated the selection process of suitable criteria as multi-criteria decision analysis (MCDA); specifically, analytical hierarchical process (AHP), multi-attribute utility theory and outranking. Outranking methods are considered appropriate for connecting spatial data with practical use cases. In these methods, criteria weighting are determined based on the judgment of the stakeholders, rather than the data processing. Hu and Gong [88] further proposed a Data Envelopment Analysis (DEA) based framework to integrate judgment from both stakeholders and data processing teams. Nevertheless, these studies only address a few use cases, further exploration is necessary.

## 4. Discussion: Future Directions on Managerial Perspective

Our analysis demonstrates sustained and widespread growth in research on the topic of deploying spatial data for coastal community resilience from a managerial perspective. All told, the articles reviewed identified the significant potential of different kinds of spatial data and their applications in decision-making in coastal resilience. However, while most studies emphasized on leveraging the use of spatial data in coastal hazards from a technical perspective, the study on the managerial perspective is sparse. According to Cash et al. [90], it was pointed out that in practice, decision-making is still hindered by lack of coordination, poor information flow, and the inability of the disaster manager to validate and process accurate information within a realistic time frame. To this end, the inconsistency and limitations in existing literature in deploying spatial data in coastal hazard decision-making may be viewed as a starting point, therefore, we argue that these limitations, in large part, help establish a framework for a future research agenda.

### 4.1. Formulate the Problem

The coordination between data providers and decision-makers are restricted due to the nature of these two parties. Decision-making is a rational managerial process to select among alternative operations and to arrange them in a logical order. The decision-making unit is either the location or the operation. On the other hand, the end products of information processing have strong potential but are not yet ready to use in decision-making. In disaster response, the usefulness of information is no longer determined by the performance of the algorithm, but a fuzzier criterion: how the processed information from these tasks supports decision-making. In the literature, numerous studies are investigating how to model the decision-making’s need from the processing goals [91,92,93,94,95,96]. For instance, Timmerman, Boer [91] clarified the process and participant for the process of identifying information need. In another example, Kuhlthau [94] proposed that information seeking should be based on the user’s perspective. All studies have profound influences in providing a theoretical background in enhancing the information coordination and collaboration, yet application-wise, this remains unaddressed [91].

### 4.2. Prioritize the Mission

Decision-making during a disaster environment has multiple objectives [97,98]; it involves varying data processing tasks and integrates multiple algorithms. For instance, during disaster response, humanitarian relief often consists of a series of tasks such as minimizing human suffering and death [71,99], designing routes for search and rescue (SAR) operations [63,100], allocating disaster relief resources, and assessing the risk of critical infrastructures to prevent secondary hazards [101,102,103]. In addition, collaboration and coordination between governmental agencies, relief organizations, and commercial companies remain problematic due to conflicting missions [46]. Nevertheless, there is limited research in identifying the importance of each disaster mission [91,93,104] and prioritizing the disaster operations [105] accordingly to maximize decision efficiency.

### 4.3. Identify the Information Salience

To support evidence-based decision-making, it is essential to ensure that information supplied by the data processing team is tailored to the needs of users [92,95,106]. Salience deals with how relevant and usable information is to decision-making bodies [107]. Salient information has repeatedly been identified as essential in evidence-based decision-making [93,108,109]. It is typical because there exists overwhelming data that may not be relevant or useful. In the meantime, spatial data acquisition will expand size and scope in the coming years [110]. Without clearly specifying the salience of different information, data suppliers could generate an excessive amount of data that might fail to provide timely and relevant information. Ward, Loftis [111] described such a situation as “data-rich but information-poor syndrome”. Even after three decades, this syndrome still exists. Identifying the salience of information remains challenging [95]. 

## 5. Conclusions

Disasters are increasingly observed and monitored using a loosely coupled network of geospatial sensors. The use of spatial data for coastal community resilience applications has diversified as a consequence of the increasing availability of data, and extensive development in the data processing. Overall, this study aims to provide insight into ways of deploying spatial data in coastal hazard decision-making. 

Spatial data have proven to offer a variety of opportunities to enhance community resilience applications. GIS data have been expanded as an integrated information system and decision support tool for disaster response. Crowdsourcing and remote sensing data play two complementary roles. Crowdsourcing applications enable a bottom-up “voice” channel from “citizens as sensors”. On the other hand, remote sensing can objectively record the morphology change of the earth’s surface and serve as a primary resource base map as well as provide rich-in-detail layers. We conducted a thorough literature survey for these three types of spatial data and summarized their roles and limitations.

While existing literature on spatial data exploits the potential of spatial data through developing complicated algorithms, our review analysis suggests that such technical solutions may not address practical needs as computational resources and that time is highly constrained with disaster data and the processing algorithms being more abundant than required. This finding has suggested that a managerial solution may be good complementation to step in to leverage spatial data in coastal hazards’ applications [12,88]. 

Therefore, rather than investigating the technical perspective of spatial data, it is deemed to incorporate the managerial perspective as the second dimension to improve the practical use. In this systematic review, we provide an analysis of what spatial data can do and what it cannot do, and based on this analysis, outline missing pieces that future research can take to improve the value of spatial data in coastal hazard operations. 

## Figures and Tables

**Table 1:** Extraction Form.

Category	Extraction
Authorship, publication year, title, journal	The basic information was extracted from the title page.
Type of study	We used four classifications extracted from the abstract: review, framework, analysis without practical use cases, analysis with practical use cases, and the information was extracted from the abstract.
Type of spatial data	LiDAR, imagery, crowdsourcing, GIS. The information is extracted from the abstract.
Outcomes	We extracted both the potentials and the limitations of spatial data from the full text.

**Table 2:** Summary of the availability of data.

Category	Type of Data	Data Source
GIS	Demographics	US Census Bureau (www.census.gov)
	Elevation Data	USGS (www.usgs.gov/products/data-and-tools/gis-data), DATA.GOV (catalog.data.gov); Open Data; Houston; NYC Open Data
	Transportation Network	TIGER/Line (www.census.gov), State Geography Information Network(e.g., NJGIN)
	Municipality Boundary	TIGER/Line (www.census.gov), State Geography Information Network(e.g., NJGIN)
	Hospitals and Medicare facilities	DHS Homeland Infrastructure Foundation-Level Data (HIFLD)
	Flood Zone	FEMA National Flood Hazard Layer (NFHL), FEMA Flood Insurance (NFIP), USGS (https://www.usgs.gov/products/data-and-tools/gis-data)
	Other Web Services	Google Map, Microsoft Bing, OpenStreetMap
Crowdsourcing/User-Generated Information	Micro-blogs	Twitter, Twitter, Blogger, WordPress, Facebook
	Discussion forums	Quora, Reddit
	Digital content sharing platforms	Flickr, Instagram, Pinterest, YouTube, Bilibili
	Social gaming sites	Gree, Mobage, Zynga
	Social networking sites	Facebook, Google+, LinkedIn, Mixi, Orkut
Remote Sensing	Aerial photographs	USGS National Assessment of Coastal Change Hazards (NACCH) project
	Satellite Imagery	NASA Earth Observatory, Google Imagery
	LiDAR	Airborne (NOAA Digital Coast https://coast.noaa.gov/digitalcoast), Mobile LiDAR (2012 Rutgers Post-Sandy Mobile LiDAR,2014–2016 Rutgers Ocean County Mobile Lidar, 2017 Rutgers Post-Harvey Mobile Lidar, [20]) Terrestrial LiDAR(USGS [20])

**Table 3:** Summary of different spatial data in addressing different dimensions of community resilience.

Category	Description	Built-Up	Environment	Social and Economic
GIS	Decision support tools [37]	Building polygons, Critical infrastructure	SLOSH: Sea, lake, and overland surges [2,39], Terrain and Wind Load Modeling (HAZUS) [40]	Damage and Loss estimation (HAZUS) [41,61], Demography, etc.
Crowdsourcing (User-Generated Information)	Citizens as sensors [45,46]	-	-	Human activity pattern [38], Sentiment analysis [17,49], Crisis communication [50];
Remote Sensing	Primary source and base map: Imagery [21]	Building [57], Infrastructure [22]	Sediment plume [53], Land surface [22,54,55,59]	Property [60]
	Rich-in-detail layers: LiDAR	Building [57,58,62], Infrastructure [63], Street furniture [64,65], Hydraulic feature [20,66]	Land surface [67], Forest and trees [68,69], Dune and beach [70]	Population [71]

**Table 4:** Summary of techniques, strengths, weaknesses, and gaps in decision support.

Category	Techniques	Strengths	Weaknesses	Gaps in Decision Support
GIS	Exploratory data analysis [43], Spatial Database [35], Operational Models [33,85], Communication [46], Hazard modeling [39,41], Decision Support [35,36,37]	Prevailing platform for spatial data storage and integration [33,37,42,46]	Deficiency in the data [34], Error propagation [78],	Adaptability to decision criteria [82,83]
Crowdsourcing (User- Generated Information)	Major Communication Channel [15,46,50,51], Sentiment Analysis [17,49], Prediction [16,80]	Opportunity to be Spatial Decision Support Systems (SDSS) [15,36,80]	Unavailability of training dataset [80], Credibility [19], Spatial Registration [86]	Timely issues [50]
Remote Sensing	Situation Awareness (Hazard Mapping) [56,59], Quantify Detailed Change Information [76,77], Quantify Detailed Information [21,62,68], Quantify Physical Geographic Phenomena [24,53], Vulnerability Analysis [32,58,74,75], providing insights into management, policy, and science.	Visualization tool for situational awareness and support rapid damage detection through change detection [62,76,77], potential to identify damage details [62,72]	Resolution [57,79], Accuracy [67,87], Computation expensive [81], Human Interaction [84]	Adaptability to decision criteria [12,88], Timely issues [88]

## Data Availability

Data sharing not applicable.
